# Using a Community Workshop Model to Initiate Policy, Systems, and Environmental Change That Support Active Living in Indiana, 2014–2015

**DOI:** 10.5888/pcd14.160503

**Published:** 2017-08-31

**Authors:** Peter J. Fritz, Kim Irwin, Lindsey Bouza

**Affiliations:** 1 Indiana State Department of Health, Indianapolis, Indiana; 2 Health by Design, Indianapolis, Indiana

## Abstract

**Background:**

Engaging in regular physical activity reduces the likelihood of developing chronic diseases. A community’s rates of physical activity are directly connected to its built environment characteristics, which correspondingly affect the chronic disease prevalence of its population. Community planning and design interventions can increase levels of physical activity and reduce chronic disease rates by identifying and removing environmental and policy barriers that may hinder active living.

**Community Context:**

Community stakeholder groups of various sizes and in various settings in Indiana are beginning to make changes to their policies, systems, and environments to increase levels of physical activity for residents.

**Methods:**

We conducted day-long active living workshops in cities and towns in Indiana to help organize and support public officials, community-based organizations, and advocates in their efforts to promote policy, system, and environmental (PSE) changes that lead to more active communities.

**Outcome:**

We found that following a consistent process of holding a community workshop and then conducting ongoing follow-up activities led to PSE changes within 1 year. Communities that hosted active living workshops created identifiable changes by supporting active living goals through policy adoption, the creation of new advisory committees, and new local funding allocations.

**Interpretation:**

The collaborative approach in the workshop provides a successful model for communities to build capacity to implement PSE strategies that support active living. This method requires various community stakeholders to work closely together, using a shared approach to make changes that would be difficult to achieve if they were working independently.

## Background

Participating in regular physical activity provides many health benefits, including the reduction of chronic diseases. Strong scientific evidence shows that, as opposed to inactive people, physically active people have lower rates of various chronic diseases, including multiple types of cancers, type 2 diabetes, and heart disease (1).

A community’s built environment characteristics and the rates of physical activity and chronic diseases among its residents are directly connected (2). By identifying and removing environmental and policy barriers that hinder active living, community planning and design interventions can increase levels of physical activity and reduce levels of chronic disease among residents (3).

Active living is defined as a way of life that integrates physical activity into everyday routines, such as walking to the store or bicycling to work (4,5). The Community Preventive Services Task Force recommends the creation of or enhanced access to places for physical activity because of strong evidence of the effectiveness of these places in increasing physical activity and improving physical fitness (6).

Studies show that communities supporting and promoting active living exhibit higher levels of both leisure-related and transportation-related physical activity. However, in many settings, environments that support active living do not typically occur without deliberate intervention through community planning and design efforts (7).

Many communities in Indiana struggle to begin planning and designing their communities to promote active living. Community leaders, advocates, and public health professionals are often not familiar with communitywide approaches to identifying opportunities for active living. It is usually necessary to provide leaders with assistance in facilitating and understanding the process of collectively supporting and promoting active living in their communities.

## Community Context

Indiana’s population is estimated by the US Census Bureau to be 6,619,680 as of July 2015 (8). In Indiana, only 44.1% of the adult population complete the recommended 150 minutes of physical activity per week (9), and only 25.3% of the youth population meet the recommended minimum of 60 minutes of physical activity per day (10). Overweight and obese adults in Indiana comprise 66.5% of the population, making Indiana the 15th most obese state in the nation (11).

Since the 1950s, communities throughout Indiana have developed without infrastructure that supports active living. From the 1950s through the 1990s, many Indiana residential and commercial developments were built without sidewalks, safe pedestrian crossings, or provisions for bicycles. This lack of safe pedestrian, bicycle, and transit options is a major barrier, severely limiting the options of Indiana residents trying to live more active lives.

Communities of various sizes and in various settings across the state are organizing stakeholder groups and initiating collaborative processes designed to increase levels of physical activity for residents. Much of this work began in 2010 with the preparation of Indiana’s Comprehensive Nutrition and Physical Activity Plan (12). This plan contains community objectives that support the active living workshop approach. The goals and objectives contained in the 2010 plan were used as a basis to apply for enhanced funding from the Centers for Disease Control and Prevention (CDC) as a means to support the active living workshop approach during the 5-year funding cycle. By providing technical assistance in assessing community physical activity policy, this approach meets an identified need in communities (13).

The objective of active living workshops and follow-up activities as public health interventions is to enable local stakeholders to understand challenges that community members face while trying to live more active lives. Workshops and follow-up activities should also provide guidance and technical assistance in addressing those challenges. The objective of community engagement efforts is to involve local citizens in educational and experiential learning activities, including presentations, walk audits, suitability mapping exercises, and exercises to identify and prioritize active living issues and action steps.

Outcomes of interest for community engagement efforts included the establishment of new active living advisory groups in communities. These groups assisted in the adoption of active living policies and programs, helped create changes to systems, and supported the construction of active living projects after completing their workshops (Table).

**Table:** Selected Workshop Locations With Short-Term Outcomes and Long-Term Results, Indiana, 2014–2015

Community	Population (2010)	Short-Term Outcomes	Long-Term Results
Lebanon	15,792	Completed bicycle and pedestrian master plan; secured $100,000 for Safe Routes to School, sidewalks; secured $100,000 budget line item for active living; enforced installation of bicycle racks with new development	Formed active living steering committee
Frankfort	16,422	Secured commitment to build bump-outs; installed bicycle racks; installed new signage for smoke-free parks, playgrounds; developed new shared-use policies and revised school wellness policy; secured active living and health references in comprehensive plan	Pursued local and grant funding; increased participation and strengthened existing partners
Batesville	6,520	Participated in first Walk to School Day; completed application for funding for bicycle lot; held Velo in the Ville event	Submitted grant applications to fund active living initiatives
Williamsport	1,898	Secured $2,500 for trail development; held bicycle rodeo, community ride and education activities; created Facebook page	A small core group has begun to meet
Madison	11,967	Worked with school corporation to fabricate bicycle racks; secured funding for bicycle and pedestrian master plan	Developed and implementing a vision and action plan; meeting quarterly
Hendricks County	145,448	Analyzed data on bicycle and pedestrian crashes	Helped build better interdepartmental and interagency relationships; improved communication; connected to community health improvement plan
Anderson	56,129	Installed wayfinding; made spot improvements to sidewalks, intersections; developed pilot Safe Routes to School programs at 2 elementary schools; planned road diet projects	Helped to start/expand community conversations about active living, walking, and bicycling
Bloomington	80,405	Held Open Streets and ShareFest events; held public education program on volunteer driver programs; included broader audience and held focus groups as part of parks and recreation master planning efforts	Expanded active living coalition membership; led to greater coordination with other groups
Decatur	9,405	Developed pilot pedestrian alley project; secured community foundation funding to create bicycle racks; attained land for riverfront development	Funded and hired full-time community coordinator; improved networking and collaboration among community partners
Pendleton	4,253	Secured funding for bicycle and pedestrian master plan; developed story map for Safe Routes to School project	Strengthened relationship, partnership with school corporation

## Methods

The workshops were conducted by staff members from the Indiana State Department of Health’s (ISDH’s) Division of Nutrition and Physical Activity (DNPA), and by Health by Design (the organization that ISDH hires to help lead the workshops). We conducted 15 active living workshops in communities in Indiana in 2014 and 2015 to help organize public officials, staff members of community-based organizations, and advocates in their efforts to promote policy, system, and environmental (PSE) changes that lead to more active communities. Funding paid for staff members of Health by Design and ISDH to assist with the workshop planning, facilitation, and follow-up activities. No funding was provided directly to the communities to facilitate the workshops or for implementation activities. The workshops were funded with 1305 funds (State Public Health Actions to Prevent and Control Diabetes, Heart Disease, Obesity and Associated Risk Factors and Promote School Health Cooperative Agreement) from CDC. The workshops were conducted in communities across Indiana. Populations of host communities ranged from 1,898 to 145,448 in both rural and urban settings. The average host community’s size was 34,824.

The workshops followed a prescribed approach that began with a competitive application and ended with the submission of a success story 1 year after the workshop (Appendix). Communities selected to host a workshop exhibited a readiness to proceed and a leadership structure that supported follow-up activities. Communities not selected either submitted incomplete applications or failed to illustrate their readiness to host a workshop. The approach used in conducting the active living workshops and associated activities was the result of lessons learned from 25 workshops on related topics conducted by ISDH DNPA before 2014. We learned from prior workshops that immersive, hands-on activities, when combined with verbal presentations and small group discussions, resulted in high evaluation ratings from attendees. We also learned that extensive preworkshop planning and coordinated follow-up led to better long-term results. We funded 15 active living workshops during 2014 and 2015, part of the 5-year 1305 funding cycle.

### Preworkshop selection and planning: before the workshop

An annual call for applications was advertised throughout the state near the end of each year to notify community leaders of the schedule to submit applications to host a workshop. Fifteen to 20 applications were submitted by communities each year during the funding cycle for the 5 available workshop slots. A selection committee — comprising representatives from the IDSH and Health by Design — used an objective selection process to review the applications on the basis of each applicant’s response to 10 questions. The 5 most qualified applicants were chosen to host a workshop each year.

Each community chosen to host a workshop was notified of its selection. An initial coordination conference call was scheduled to begin the process of organizing the workshop. Communities that were not chosen were offered consultations to discuss how they could prepare a successful application in the future. Two communities that were not selected for workshops requested follow-up consultations during this period.

The initial workshop coordination call included key community leaders who were the point of contact throughout the workshop and the year-long follow-up activities. The workshop agenda and required follow-up activities were discussed in detail. A list of potential groups to attend the workshop was provided to organizers to ensure that a broad representation of local leaders attended the workshop.

A preworkshop site visit was conducted with 6 of the 10 communities to view the walk audit route, finalize the design visualization location, and see the room where the workshop was to be located. Follow-up conference calls with the host community were conducted if a site visit was not possible. The conference calls provided an opportunity to coordinate various details of the workshop.

Before the workshop, the facilitation team and local organizers conducted promotional activities to maximize attendance and ensure participation from community representatives. The facilitation team prepared a workshop flyer and press release and provided it to the local organizers for distribution in the community. The facilitation team provided guidance on how to promote the workshop to potential attendees. An online signup page was also provided to allow workshop attendees to preregister. The facilitation team periodically reviewed the registration list and provided guidance to the local organizers if key community leaders had not registered. The preregistration process has been effective in monitoring the local promotional efforts of the workshops — we have not had to cancel any of the workshops because of low numbers of preregistered attendees. Workshops were typically scheduled during the warmer months of May through October in Indiana to allow for comfortable conditions for the walk audit.

### Workshop activities: a detailed look

The day-long workshop began with presentations that outlined the connection between public health and the built environment, as well as PSE change. Local, regional, state, and national data on public health indicators were presented to help attendees understand the challenges communities face when becoming healthier. Mayors, hospital administrators, and other elected and appointed officials typically spent the entire day at the workshop.

A guided walk audit was conducted during the morning. The walk audit route was typically 1 half-mile to 1 mile long, determined by the location of the workshop, and began with a brief presentation on elements that support a good walking environment. The intent was to enable attendees to see the built environment through the eyes of a pedestrian in various settings, including residential and commercial areas.

The walk audit was led by a facilitator who stopped at various points along the route to identify features that support walking and those that are barriers to walking. Attendees were asked for their observations of conditions along the route. When a member of the community with a disability participated in the walk audit, he or she provided a good perspective and a better understanding of the challenges that people with disabilities face in negotiating the built environment. Many attendees responded in the workshop evaluation that the walk audit provided a new appreciation of the barriers that prevent all members of the local community from living a more active life.

**Figure 1:**
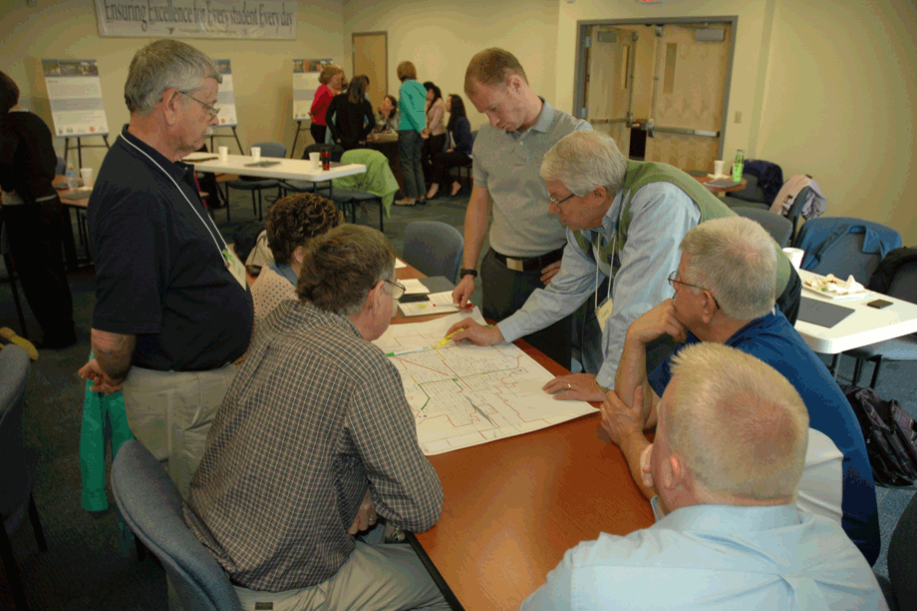
Workshop mapping exercise, using a community workshop model to support active living in Indiana, 2014–2015.

An active living suitability mapping exercise was conducted after lunch, using maps of the community prepared by the local host (Figure 1). Small groups (8 attendees per map) noted local destinations, as well as dangerous intersections and the level of suitability of roadway corridors for walking and bicycling. Each small group presented the results of their map to the entire group. The maps were photographed and included in the summary report for the workshop.

**Figure 2:**
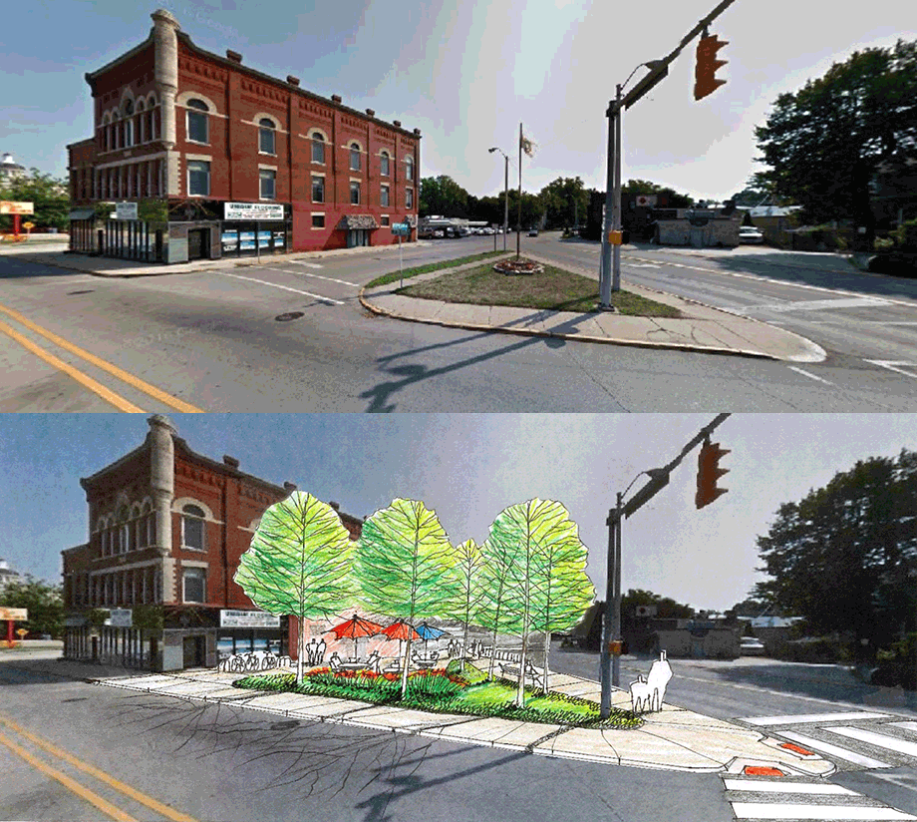
Design visualization sketch, using a community workshop model to support active living in Indiana, 2014–2015.

After the mapping exercise, we gave a presentation on best practices for planning and designing for active living. The presentation included best practices organized under the “5 Ps” of active living: policies, programs, plans, projects, and performance measures. A design visualization sketch, which illustrates a before-and-after design solution that enhances bicycle or pedestrian safety in a previously identified area in the community in need of a physical improvement, was also included in this part of the workshop (Figure 2).

The workshop concluded with an issue identification and prioritization exercise held in small groups. Tables of 8 attendees self-selected an active living topic to discuss. Topics have included walking, bicycling, land use and public spaces, parks and recreation, schools, and transit. Each group discussed both short-term and long-term ideas to address active living in the community and reported their findings to the larger group. The short-term and long-term strategies identified by each group were posted on a wall in the room. The workshop concluded with each attendee voting for the 3 issues they consider most important in both the short-term and long-term categories, using a dot voting method. This exercise was useful for visualizing and identifying consensus among the group on the various priorities discussed.

### Workshop follow-up: 1 year postworkshop

For all workshops, a summary document was prepared and sent to each attendee by email. This document included links to electronic copies of the presentations, a summary of the activities of the workshop, a description of the walk audit, the before-and-after design visualization sketch, a summary of the mapping exercise, and a summary of the issue-identification exercise, with the priority issues identified through the dot voting. Links to sources of additional information were listed under each of the issues that the groups identified in the small group session during the workshop.

Three months after the workshops, each community prepared and submitted an action plan to the state workshop team, using a template provided to assist with its preparation. After 9 months, a progress report was completed using an online survey tool. Finally, the local workshop team wrote and submitted a success story to document the outcome of the year-long effort. Often, the local community workshop team leaders created a small working group to assist in preparing the action plan and the success story. Many communities dedicated staff time and funding to assist with the preparation and implementation of the action plan and the success story.

The effectiveness of the workshop was evaluated at several points during the process. At the end of each workshop, attendees completed an evaluation survey. The action plan submitted by the local organizer 3 months after the workshop was a good way to assess the results of the prioritization exercise. A progress report form was sent to the local organizer 9 months after the workshop to gather data on their progress in implementing their action plan.

## Outcome

Outcomes of interest focused on community accomplishments within 1 year of conducting the active living workshops. Communities established active living advisory groups, adopted active living policies and programs, created changes to systems, leveraged new funding, and constructed projects after completing their workshops (Table).

Many communities experienced quick results after hosting a workshop (Table). Some communities organized new committees to guide change; others adopted new policies. A few allocated new funding to implement the ideas that emerged from the workshops. One community created a new Active Living Committee comprising the attendees of the workshop. The committee helped guide the spending of $100,000 that was allocated by the city council (after conducting the workshop) for active living improvements in the city. That same community created a bicycle festival and parade that was conducted by the workshop organizers to promote active lifestyles. After their workshop, 1 county allocated $2,500 to help plan activities in support of a new regional trail. One organizer said of the workshop, “[It] was one of the most important events that the Adams County Winning With Wellness Coalition has ever done for the improvement of our community.”

Some communities found that the workshop increased the community’s capacity for collaboration and identified challenges they were facing. Unfortunately, an organizer of the Shelbyville workshop was struck by a vehicle while walking near downtown the day after the workshop. The workshop organizers immediately used presentation materials from the workshop to make a case to the Indiana State Department of Transportation (INDOT) for emergency pedestrian-safety improvements at the intersection of the crash.

Other communities realized the challenges they face in facilitating change on state highways running through their communities. Attendees at most workshops expressed difficulty in understanding the complex funding, regulatory, and development approvals necessary to make active living improvements along these corridors. We now ask for a regional representative of INDOT to participate in all our workshops to address these important concerns.

The workshop provides a unique opportunity for local and regional public health practitioners to communicate with community members. For many attendees, the workshop is the first opportunity they have had to work with one another. The Purdue University Extension Service recently hired more than 40 county-based community wellness coordinators to implement PSE changes throughout Indiana. The workshops have provided an opportunity for the newly hired community wellness coordinators to engage with their communities. The Purdue University Extension Service office is organizing their own active living workshops throughout the state to engage their community wellness coordinators with local stakeholders.

We found that using a consistent process — holding a community workshop and then conducting ongoing follow-up activities — led to PSE changes within a year. Our findings indicate that the workshops created identifiable changes that support active living in communities that hosted them, resulting in policy adoption, the creation of new advisory committees, and allocation of new local funding.

## Interpretation

The active living workshops in Indiana are helping local communities implement PSE changes to promote the levels of active living they desire. One of the keys to success in this type of intervention is to have paid staff members on the statewide workshop facilitation team who have the capacity to organize and follow up with the communities that host a workshop.

We found that the consistent, ongoing communication and reporting required for this program creates greater outcomes than a workshop without any required preliminary planning and ongoing follow-up activities. Funding time for the facilitation staff members to oversee and manage the process before, during, and after the workshop is critical to the overall success of the program. The year-long follow-up process after the workshop is consistently effective in implementing the local action plans that communities have created.

We found that the communities involved desire an ongoing forum to discuss their efforts in promoting active living. To respond to this need, we invited community representatives who hosted a workshop to a peer exchange meeting at the 2016 statewide walking and bicycling summit. The intent of this exchange was to enable communities to share their successes and challenges in implementing active living initiatives. Representatives of communities scheduled to host a workshop in the future also attended the peer exchange meeting. The meeting provided a valuable forum for both existing and future grantees under this program to plan and implement their active living PSE strategies.

Some communities face challenges in preparing a winning application to host a workshop. Many communities with the greatest need have the least capacity to prepare an application that meets the requirements of being selected. We facilitated a half-day training session in a community that was not selected to host a workshop (although they had applied twice). The intent of the training was to increase the capacity of the community leaders to prepare a successful application in the future. The community submitted an application the following year to host an active living workshop, and it was selected. We are exploring additional half-day trainings to assist communities that need assistance preparing a successful application.

The use of a community workshop combined with structured follow-up activities provides an effective framework for implementing PSE changes to support healthier communities. The collaborative approach among various groups within the workshop setting provides an accepted model for community leaders to work toward long-term implementation of PSE strategies. This work requires alignment and coordination of various community stakeholders, offering a shared approach to accomplishing change that would be unlikely if they were working independently.

## Appendix: Active Living Workshop Timeline, Showing Workshop Tasks and Months to Complete, Using a Community Workshop Model to Support Active Living in Indiana, 2014–2015

This file is available for download as a Microsoft Word document.
