# Supplementary material for: Using a Community Workshop Model to Initiate Policy, Systems, and Environmental Change That Support Active Living in Indiana, 2014–2015
Source: Prev Chronic Dis. 2017 Aug 31;14:E74. doi: 10.5888/pcd14.160503 (PMC5580728; doi:10.5888/pcd14.160503)
Supplement: Supplementary file 1 [file 16_0503Appendix.docx]

| **WORKSHOP TASK** | **MONTHS** | | | | | | | | | | | | | | | | | | |
| --- | --- | --- | --- | --- | --- | --- | --- | --- | --- | --- | --- | --- | --- | --- | --- | --- | --- | --- | --- |
|  | **1** | **2** | **3** | **4** | **5** | **6** | **7** | **8** | **9** | **10** | **11** | **12** | **13** | **14** | **15** | **16** | **17** | **18** | **19** |
| **Pre-Workshop Selection and Planning** | | | | | | | | | | | | | | | | | | | |
| Call for Applications |  |  |  |  |  |  |  |  |  |  |  |  |  |  |  |  |  |  |  |
| Review and Select Host Communities |  |  |  |  |  |  |  |  |  |  |  |  |  |  |  |  |  |  |  |
| Conduct Pre-workshop Coordination Call |  |  |  |  |  |  |  |  |  |  |  |  |  |  |  |  |  |  |  |
| Workshop Promotion and Registration |  |  |  |  |  |  |  |  |  |  |  |  |  |  |  |  |  |  |  |
| **Workshop Activities** | | | | | | | | | | | | | | | | | | | |
| Conduct the Workshop |  |  |  |  |  |  |  |  |  |  |  |  |  |  |  |  |  |  |  |
| **Workshop Follow-Up** | | | | | | | | | | | | | | | | | | | |
| Workshop Summary Document |  |  |  |  |  |  |  |  |  |  |  |  |  |  |  |  |  |  |  |
| Submit Action Plan |  |  |  |  |  |  |  |  |  |  |  |  |  |  |  |  |  |  |  |
| Submit Progress Report |  |  |  |  |  |  |  |  |  |  |  |  |  |  |  |  |  |  |  |
| Submit Success Story |  |  |  |  |  |  |  |  |  |  |  |  |  |  |  |  |  |  |  |
